# Improving Adherence to the Mediterranean Diet in Early Pregnancy Using a Clinical Decision Support System; A Randomised Controlled Clinical Trial

**DOI:** 10.3390/nu15020432

**Published:** 2023-01-14

**Authors:** Panos Papandreou, Charalampia Amerikanou, Chara Vezou, Aristea Gioxari, Andriana C. Kaliora, Maria Skouroliakou

**Affiliations:** 1 Department of Nutrition, IASO Hospital, 15123 Athens, Greece; 2 Department of Dietetics and Nutritional Science, School of Health Science and Education, Harokopio University, 17671 Athens, Greece; 3 Department of Nutritional Science and Dietetics, School of Health Science, University of the Peloponnese, Antikalamos, 24100 Kalamata, Greece

**Keywords:** pregnancy, Clinical Decision Support System, Mediterranean diet, MedDietScore, clinical trial

## Abstract

Prenatal health is important for both mother and child. Additionally, the offspring’s development is affected by the mother’s diet. The aim of this study was to assess whether a Clinical Decision Support System (CDSS) can improve adherence to the Mediterranean diet in early pregnancy and whether this change is accompanied by changes in nutritional status and psychological parameters. We designed a three month randomised controlled clinical trial which was applied to 40 healthy pregnant women (20 in the CDSS and 20 in the control group). Medical history, biochemical, anthropometric measurements, dietary, and a psychological distress assessment were applied before and at the end of the intervention. Pregnant women in the CDSS group experienced a greater increase in adherence to the Mediterranean diet, as assessed via MedDietScore, in the first trimester of their pregnancy compared to women in the control group (*p* < 0.01). Furthermore, an improved nutritional status was observed in pregnant women who were supported by CDSS. Anxiety and depression levels showed a greater reduction in the CDSS group compared to the control group (*p* = 0.048). In conclusion, support by a CDSS during the first trimester of pregnancy may be beneficial for the nutritional status of the mother, as well as for her anxiety and depression status.

## 1. Introduction

The maternal diet during pregnancy has been linked to the health of both the mother and her offspring [1,2]. Adoption of a Western dietary pattern during pregnancy, i.e., low consumption of fruits, vegetables, whole grains, fish/seafood, and low-fat dairy products in combination with high intake of sugar and fats, has been associated with an increased risk of developing gestational diabetes [3,4] and having preterm birth [5,6]. Furthermore, offspring run a high risk of developing type 2 diabetes mellitus [7,8,9,10], cardiovascular disease [10,11], atopic dermatitis, and allergies [12,13,14] during childhood and adulthood as well. Given that diet constitutes a modifiable factor, it is crucial for healthcare professionals to intervene to improve the health of both the mother and the child. Targeting the adoption of the Mediterranean diet (MD) pattern would be a beneficial practice for their health [15].

Studies have established that MD promotes maternal health during pregnancy and child health after delivery [15,16]. MD entails a high intake of fruits, vegetables, whole grain cereals, legumes, fish, nuts, and olive oil as the main source of fat [17]. A useful tool developed by Panagiotakos et al. in 2006 [18] for evaluating MD adherence is the MedDietScore. It incorporates the substance components of MD, records them through food diaries and food frequency questionnaires, and produces a score that, within specific ranges, has been associated with beneficial effects on human health [19]. Investigating the effects of dietary patterns and not only of particular nutrients, the MedDietScore provides information for appropriate diet recommendations and supplementation in pregnancy [20].

Clinical Decision Support Systems (CDSS) date back to the 1970s [21], but the term was unanimously defined in 1991 as “active knowledge systems that use two or more items of patient data to generate case-specific advice” [22]. Since then, the increasing progress of computer and software technology has enabled the analysis of numerous and complex parameters, and their outputs can be obtained either through portable devices such as smartphones and laptops or other devices such as biometric monitoring and wearable health technology, or they can be linked into electronic health record databases [23]. CDSSs have been developed to facilitate healthcare professionals’ recording health data for their patients, combiningit with consolidated clinical data to make accurate diagnoses, implementing guidelines, andpredicting pharmaceutical contradictions [24]. The CDSSs have been used in clinical trials for promoting healthy diets and lifestyle practices.

Within this scope, we have developed a CDSS to enable individuals to follow diet and lifestyle instructions from home, avoiding visits to the healthcare setting, with the intention of minimizing the risk of COVID-19 infection. The present CDSS was developed by our research team in 2016, and ever since, it has been applied in clinical practice to assist dieticians in the nutritional care process, such as nutritional screening and monitoring. The CDSS allows patients to visit their personal dietary regimen at any time and to record and monitor their own progress, e.g., goals of body weight, physical activity, and consumption of fruits, vegetables, and legumes, via a login password [25,26,27,28]. Records can automatically be made available to the attending health care professional (e.g., physician, dietician), who will in turn assess patient progress and nutritional status. Further on, the CDSS intends to ease the collaboration of healthcare professionals (e.g., gynecologist, obstetrician, dietician, health psychologist) by improving a broad range of clinical practices, such as patient assessment, monitoring, and lifestyle counseling, in a cost-effective context. Upon the strict adjustments in healthcare units to limit individuals’ COVID-19 exposure, the application of CDSS could be a desirable way to upgrade access to health provision.

In the present study, we hypothesised that the incorporation of CDSS into dietary practices ameliorates nutritional status and reduces health-related anxiety and depression among pregnant women. Therefore, we aimed to investigate whether:

(a) MD adherence increases in healthy pregnant women with the support of CDSS compared to controls who receive standard guidelines for pregnancy;

(b) Pregnant women intervened by CDSS have an improved nutritional status in terms of anthropometry, dietary intake of nutrients, and biochemical profile, as well as a better psychological state (depression and anxiety) than pregnant controls.

To our knowledge, this is the first study to investigate the effectiveness of CDSS in improving MD adherence among pregnant women.

## 2. Materials and Methods

### 2.1. Ethics and Participants

Ethical approval was obtained from the Iaso Hospital Institutional Review Board (approval code #b31052019). Apparently healthy pregnant women who were outpatients of the IASO Maternity Hospital, Obstetrics—Gynecology (Athens, Greece), were invited to participate in the study via written announcements (posters) at the clinic facilities and the website. Women willing to participate were informed in detail about the aims, methods, benefits, and potential harms of the study. A signed informed consent was obtained from all subjects involved, and a copy of the signed document was kept by each subject. The principles of the Declaration of Helsinki were adhered to throughout the intervention, while the ClinicalTrials.gov registry was acquired (NCT05634837). The intervention took place from May 2019 to May 2022. The inclusion and exclusion criteria are shown in Table 1.

### 2.2. Study Design

Eligible women were randomly assigned in blocks of one to either the control arm or the intervention arm. Researchers and patients were aware of the treatment allocation, except for the appointed statistician, who was blinded. The statistician applied simple randomisation through a computer-generated randomisation sequence, and the randomisation list was available only to the principal investigator.

Throughout the trial, each enrolled subject from both groups attended two personal sessions with the appointed researchers, at baseline and at follow-up (3 months later), during which anthropometry, dietary assessment, and blood withdrawal were performed. More specifically, at baseline, before the start of the trial, each participant was appointed to a well-experienced dietician. In the intervention group (the CDSS group), the dietician administered a personalised daily dietary plan based on the MD that was generated by the CDSS software, according to the participant’s needs, habits, and preferences. All components needed for the dietary plan synthesis, i.e., current and pre-pregnancy body weight, basal metabolic range (BMR), physical activity level based on the concept of metabolic equivalent of task (MET), gestational age, as well as macronutrient distribution, were calculated using the CDSS database as previously described [27]. The CDSS dietary regimen consisted of a daily eating programme that was renewed every 15 days, paired with nutritional recommendations that were in line with the “National Dietary Guidelines for Pregnancy” [29]. A representative example of the CDSS-produced dietary plan is shown in Supplementary Table S1. The women acquired individual CDSS login passwords and were trained to use the software by the appointed dieticians. They were instructed to regularly visit their CDSS account from home and track their nutritional status, regarding body weight gain and healthy eating. On a weekly basis, participants were also instructed to input a 3-day food diary in the CDSS that was made automatically available to the dieticians. Every other week, phone interviews were performed to support nutritional and lifestyle consultations. Additionally, unexpected phone calls were made to obtain 24 h dietary recalls.

Participants in the control group did not have access to CDSS and only received general lifestyle guidelines based on the “National Dietary Guidelines for Pregnancy” through phone call sessions with the dieticians every 15 days. The women in the control group were instructed to keep a 3 day food diary every week, which was sent by email to the appointed dietician. Again, unexpected phone calls were made to obtain 24 h dietary recalls.

### 2.3. Screening and Assessments

Medical history: before study initiation, the attending gynaecologist obtained a detailed medical history, including gestational age, weight gain during pregnancy, smoking, alcohol consumption, and any potential concomitant medical conditions. Dietary assessment: a semi-quantitative Food Frequency Questionnaire (FFQ), validated in the Greek population, was applied [30]. The questionnaire assessed the intake of 156 foods and beverages commonly consumed in Greece, with seven non-overlapping response categories. The frequency of consumption was determined as the number of times a food was consumed within a month in small, medium, or large portion sizes, with the help of food models and pictures of portion sizes. From each FFQ at baseline and follow-up, the MedDietScore was computed to assess adherence to the Mediterranean dietary pattern; within a range of 0 to 55, higher MedDiet scores were interpreted as having greater adherence [18]. The Nutritionist Pro™ (Axxya Systems, Stafford, TX, USA) software was used to analyse food diaries and 24 h dietary recalls for energy, macronutrient, and micronutrient intakes at baseline and at the trial endpoint (3 months).

Psychological distress assessment: We applied the Hospital Anxiety and Depression Scale (HADS), which comprises 14 self-assessed items, of which seven are related to depression and the other seven to anxiety. Each category is scored in the range of 0–21, and scores higher than “7” indicate possible cases of anxiety or depression, respectively [31].

Anthropometrics: self-reported data on body weight prior to pregnancy were collected. Current body weight (BW) and body fat mass (BFM) percentage were measured at the beginning and at the trial endpoint (3 months) with the method of Air Displacement Plethysmography for research and clinical applications (BOD POD^®^ Body Composition Tracking Systems, Life Measurement, Inc., Rome, Italy), as previously described [27]. Height was measured with a calibrated stadiometer to the nearest 0.1cm. Pre-pregnancy BMI was calculated as the ratio of weight (kg) to the square of height (m^2^) and was defined as underweight (<18.5), normal weight (18.5–24.9), overweight (25.0–29.9), or obese (≥30) according to the “WHO, Global Database on Body Mass Index” for adults [32].

Blood sample collection and analysis: standard blood withdrawal (15mL) from each participant was performed at both baseline and follow-up (3 months later) after overnight fasting. Whole blood samples were allowed to clot at room temperature for 20 min in order to isolate serum. Plasma was isolated by mixing whole blood with edetic acid (EDTA), an anticoagulant. For plasma and serum collection, blood samples were first centrifuged at 3000 rpm for 10 min at 4 °C. Samples were aliquoted and kept at −80 °C until further analysis.

Blood analyses: serum glucose, total cholesterol (T-CHOL), high-density lipoprotein (HDL), low-density lipoprotein (LDL), triacylglycerols (TG), and C-reactive protein (CRP) were quantified with an automatic biochemical analyser (Cobas 8000 modular analyser, Roche Diagnostics GmbH, Mannheim, Germany).

### 2.4. Statistical Analysis and Primary Outcomes

The primary outcome of the study was the change in Mediterranean diet adherence, as assessed by MedDietScore. Secondary outcomes included changes in anthropometric parameters, blood parameters, dietary intake, and the Hospital Anxiety and Depression Scale.

The Shapiro–Wilks test was used to determine normal distribution. Continuous variables are presented as mean plus standard deviation (SD) or median plus interquartile range (IQR) for normally and not normally distributed variables, respectively. Categorical values are expressed as counts (*n*) and percentages (%). For the comparison of means between the control and CDSS groups, we used the independent sample t-test for normally distributed variables or the Mann–Whitney U test for those that were not normally distributed. The chi-square test was used to test differences between the two groups for categorical variables. For the comparison of the means inside a group before and after the intervention, we used a paired-samples t-test for normally distributed variables or the Wilcoxon signed-rank test otherwise. For the comparison of mean changes between groups, we used a repeated-measures ANOVA, and a *p*-value <0.05 was considered significant for all tests. Significant *p*-values were corrected for multiple testing using the Bonferroni correction. All analyses were conducted using the SPSS statistical software (version 22.0).

## 3. Results

**Figure 1:**
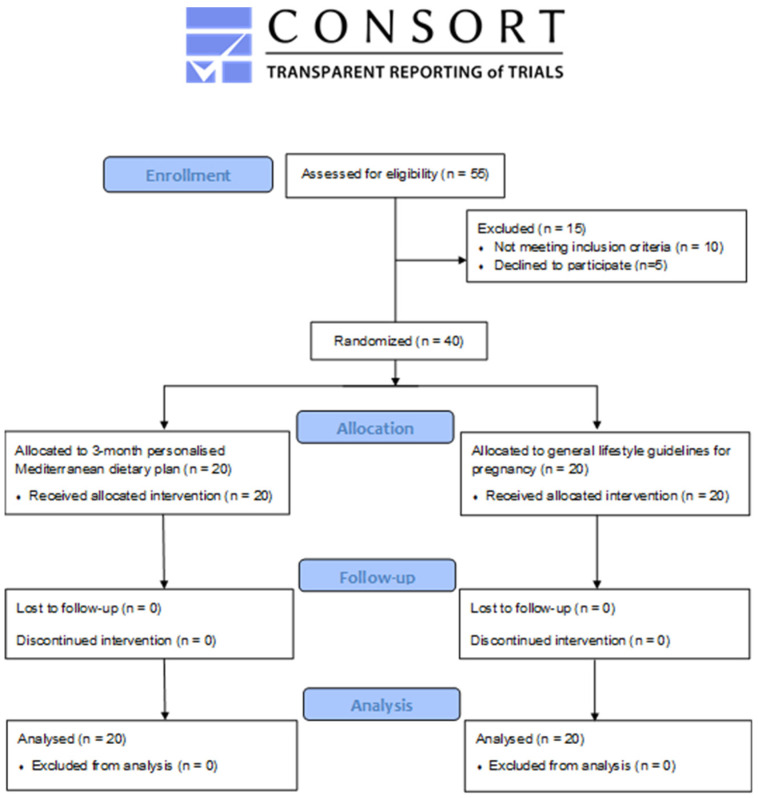
CONSORT flow diagram.

As shown in Figure 1, a total of 55 women responded to the study invitation, while 40 met the inclusion criteria and were enrolled. Participants were divided into two equivalent groups: the intervention (*n* = 20) and the control (*n* = 20) group. All the volunteers completed the study and were included in the final analysis. All participants were residents of Attica (Greece). Additionally, they were non-smokers and had ≤1 serving of alcohol per month.

At baseline, the mean age was 30.8 years. Based on pre-pregnancy self-reported body weight, 85% were classified as having a normal BMI, 12.5% as overweight, and the remaining 2.5% as underweight. Baseline characteristics of the participants are shown in Table 2. Overall, the characteristics of the participants were well balanced between the two groups. Regarding the profession, most women have been working in the private sector (32.5%) or in housekeeping (35.0%). Only ten of them smoked before pregnancy, with only three of them continuing to smoke during pregnancy. Alcohol consumption was reportedby only 5% of the participants.

The effects of intervention on anthropometry, dietary intake, and psychological distress at baseline and follow-up, as well as the comparison between the two groups, are presented in Table 3, Table 4 and Table 5, respectively.

Changes in anthropometric measurements and blood markers are presented in Table 2.Body weight increased significantly in both groups after 3 months, whereas % fat increased only in the control group (*p <* 0.001 in all cases). Total cholesterol (*p* = 0.002), LDL (*p* = 0.002), glucose (*p <* 0.001), and triacylglycerols (*p* = 0.001) decreased significantly only in the intervention group. No significant difference was observed in the two treatments when exploring the between subjects’ effect.

Changes in dietary intake after the 3month intervention are presented in Table 3. MetDietScore was higher at baseline and at follow-up in the CDSS vs.the control group (*p <* 0.001 in both cases). After the intervention, MedDietScore and fibre intake increased only in the CDSS group (*p <* 0.001 in both cases). Energy intake (*p <* 0.001) was lower in the CDSS group before the intervention. Finally, in both MetDietScore and energy intake, a significant difference was observed in the two treatments when exploring the between subjects’ effect (*p <* 0.001 in both cases).

The evaluation of psychological distress with the HADS scale is presented in Table 4. Anxiety and depression levels reduced significantly in the CDSS group (*p <* 0.001). Additionally, HADS levels were statistically significantly lower in the CDSS group at follow-up when compared with the levels of the control group at the same time point.No significant difference was observed in the two treatments when exploring the between subjects’ effect and after applying the Bonferroni correction.

## 4. Discussion

In the present 3month randomised controlled trial, we explored whether a personalised dietary regimen together with nutritional consultation supported by the CDSS, would be beneficial in improving MD adherence of healthy pregnant women. Our results indicate that the CDSS can assist clinicians to increase MD adherence during pregnancy and enhance nutritional status by increasing dietary intake of fiber. In fact, women following MD ameliorated body composition, blood lipid profile, and serum glucose at the study endpoint (3 months).

It is well established that greater adherence to the MD during gestation is beneficial for the health of both the mother and the offspring [15]. MD is considered a healthy eating pattern that provides the nutritional requirements for the prevention of obstetric pathologies, such as gestational diabetes mellitus (GDM), overweight, complications of childbirth, and preeclampsia [33,34]. Several CDSSs have been used during pregnancy either for the prediction of pregnancy outcomes or for improving medical care quality and improving adherence to clinical practices and delivery outcomes [35]. In our study, we used a CDSS in order to increase adherence to MD and reduce health-related anxiety and depression among pregnant women, an effort that has been applied for the first time in Greece.

Regarding our primary outcome, we succeeded in proving our hypothesis that the use of CDSS increases adherence to the MD, as pregnant women in the CDSS group experienced an increase in the MedDietScore at the first trimester of their pregnancy, while women in the control group who received general lifestyle guidelines did not. Similarly, the same effect of CDSS application on MD adherence was observed in other studies of our group, which included breast cancer and multiple sclerosis patients [25,27]. As mentioned previously, a significant increase in MD adherence during pregnancy has been associated with several beneficial outcomes for both the mother and the offspring. In the St. Carlos GDM Prevention Study, in Spain, 874 pregnant women who received a MedDiet-based medical nutrition therapy as part ofGDM management, women with GDM exhibited similar HbA1c levels at 36–38 gestational weeks as those of women with normal glucose tolerance (NGT) [36]. Additionally, a Mediterranean-style diet with a higher intake of nuts, olive oil, fruits, vegetables, non-refined grains, and legumes was associated with less gestational weight gain and a reduced risk of GDM [37]. Other studies have shown that higher MD adherence is associated with better sleep quality [38] and lower preeclampsia odds [34]. Regarding perinatal outcomes, adherence to the MD during pregnancy, as estimated via an a priori defined score (MDS), was associated with lower BMI, waist circumference, and systolic and diastolic blood pressure in the offspring, suggesting a cardioprotective role of the MD [39]. In the St. Carlos GDM Prevention Study, the nutritional intervention based on the MD during pregnancy was associated with a reduction in the offspring’s hospital admissions [40], and a post-hoc analysis in the same study showed that late first-trimester high adherence to the MD was associated with a lower risk of prematurity and small-for-gestational-age newborns [16]. Finally, Chatzi et al. [41] showed that children whose mothers had a great deal of adherence to theirMD during pregnancy have a reduced risk of experiencing wheeze and atopy at the age of 6.5 years.

Our study also showed improved nutritional status in pregnant women who were supported with CDSS during the first semester of the pregnancy compared to those who only received lifestyle counseling. The CDSS group increased fibre intake, while the control group did not. Additionally, a slight increase in energy intake in the CDSS group and a respective decrease in the control group resulted in a significant difference between changes at follow-up. After follow-up, the percentage of fat intake in the intervention group increased, but that of the control group remained unchanged. The mean changes in percent fat intake of the two groups were significant at follow-up. Since olive oil is abundantly used in cooking and dressing, the MedDiet is not considered a high-unsaturated-fat food pattern.

Regarding psychological status, pregnant women in the CDSS group presented a better anxiety level at the end of the trial, whereas the control group had a better depression level, although the depression category did not change in either group. Anxiety and depression during pregnancy have been associated with undesirable outcomes for both the mother and the offspring, such as shorter gestation, lower birth weight, and impaired foetal neurodevelopment [42]. Therefore, an increase in the HADS score is essential, especially during the first semester. The HADS-A has been used to measure anxiety during pregnancy [43,44]. Not many studies have examined the association between MD adherence and symptoms of anxiety and depression in pregnancy. In a secondary analysis of the GESTAFIT Trial, an intervention that investigates the effects of exercise on postpartum depression, a higher adherence to the MD during pregnancy was associated with fewer depressive symptoms and a lower risk of postpartum depression, as depicted in the Edinburgh Postnatal Depression Scale [45]. Nevertheless, several studies in the general population indicate a protective effect of MD on depression. For example, the SUN study, a large prospective Spanish study, demonstrated that adherence to the MD pattern was associated with a more than 30% reduction in depression risk [46]. Additionally, different intervention trials with the MD in patients with depression resulted in fewer depressive episodes compared to the control [47,48,49].

Potential limitations of the present study are the sample size, which is relatively small, and the use of self-reported tools such as food diaries. Therefore, results should be interpreted with caution. Nevertheless, we tried to address these limitations; we applied a computer-generated simple randomisation that was applied by a statistician who was not aware of the treatment allocation. Furthermore, all the appointed dieticians who supervised participants throughout the intervention were well experienced. They were able to identify possible disparities and seek clarifications from participants. Additionally, all tools used for nutritional and psychological status (FFQ, MedDietScore, and HADS) have been validated in the Greek population. Another potential limitation that occurs in nutritional interventions is treatment contamination in the control group. To overcome this, different dieticians were assigned for each study group. Finally, we recognise that subjects with limited computer skills would have difficulties using the CDSS in everyday life. Therefore, before the start of the intervention, all volunteers were trained to use the CDSS and were supported throughout the study.

## 5. Conclusions

In conclusion, our results support the use of CDSS during pregnancy for improving MD adherence and overall psychological distress, as evidenced by better anxiety and depression scores. More studies are needed in this field in order to establish the role of CDSS in increasing MD adherence during pregnancy and reinforce its use in nutritional guidelines during the first trimester.

## Figures and Tables

**Table 1:** Inclusion and exclusion criteria for study participation.

Inclusion Criteria	Exclusion Criteria
Adult women (≥18 years old) in the first trimester of pregnancy.	Adolescent pregnancy.
	Women in the second semester of pregnancy.
- Without pregnancy complications.	Women with pregnancy complications, e.g., infections, hypertension, pre-eclampsia, gestational diabetes.
- Without medical conditions before pregnancy.	Women with allergies or food intolerances.
- Who provided signed participation consent.	Women with pre-pregnancy chronic diseases (e.g., malignancy, cardiovascular diseases), inherited metabolic diseases, malabsorption, or cognitive disorders.
	Women with psychiatric conditions.
	Women with alcoholism or drug addiction.
	Women following a vegan or macrobiotic diet ≤ 5 years prior to intervention.
	Women with vitamin or mineral supplementation ≤ 6 months prior to fetus conception.
	Inability to read and understand the consent information.

**Table 2:** Baseline characteristics of the sample.

Characteristics	Enrolled Pregnant Participants (*n* = 40)	Control Group (*n* = 20)	CDSS Group (*n* = 20)	*p-*Value
Age (years) mean (SD)	30.8 (6.4)	29.1 (6.1)	32.5 (6.4)	0.099
Pre-pregnancy BMI (kg/m^2^) *n* (%)				
<18.5	1 (2.5)	1 (5.0)	0 (0.0)	0.549
18.5–24.9	34 (85.0)	17 (85.0)	17 (85.0)	
25–29.9	5 (12.5)	2 (10.0)	3 (15.0)	
>30	0 (0.0)	0 (0.0)	0 (0.0)	
Fat (%) mean (SD)	29.4 (6.9)	29.2 (6.9)	29.6 (7.0)	0.862
Fat Free Mass (%) mean (SD)	70.6 (6.9)	70.8 (6.9)	70.4 (7.0)	0.862
Profession, *n* (%)				0.060
Private sector	13 (32.5)	5 (25.0)	8 (40.0)	
State employee	4 (10.0)	1 (5.0)	3 (15.0)	
Housewifery	14 (35.0)	6 (30.0)	8 (40.0)	
Other	9 (22.5)	8 (40.0)	1 (5.0)	
Smoking before pregnancy, *n* (%)				0.901
No	21 (52.5)	11 (55.0)	10 (50.0)	
Yes	10 (25.0)	5 (25.0)	5 (25.0)	
N/A	9 (22.5)	4 (20.0	5 (25.0)	
Alcohol consumption				0.962
No	29 (72.5)	15 (75.0)	14 (70.0)	
Yes	2 (5.0)	1 (5.0)	1 (5.0)	
N/A	9 (22.5)	4 (20.0)	5 (25.0)	
HADS: anxiety *n* (%)				
0–7 (%)	16 (40.0)	7 (35.0)	9 (45.0)	0.807
8–10 (%)	9 (22.5)	5 (25.0)	4 (20.0)	
11–21 (%)	15 (37.5)	8 (40.0)	7 (35.0)	
HADS: depression *n* (%)				-
0–7 (%)	40 (100.0)	20 (0.0)	20 (0.0)	
8–10 (%)	0 (0.0)	0 (0.0)	0 (0.0)	
11–21 (%)	0 (0.0)	0 (0.0)	0 (0.0)	

Quantitative variables are expressed as means (SD), as all variables are normally distributed, whereas categorical variables are expressed as *n* (%). An independent sample t-test was used for the comparison of means between the control group and the CDSS group. A chi-square test was used to test differences in categorical variables. The level of significance was set at 0.05. BMI, body mass index; CDSS, clinical decision support system; and HADS, Hospital Anxiety and Depression Scale.

**Table 3:** Anthropometric characteristics and blood markers at baseline and follow-up.

Characteristics	Group	Baseline (*n* = 20)	Follow-Up (*n* = 20)	*p* ^2^	*p* ^3^
Body weight (kg) mean (SD)	control	64.3 (8.3)	67.8 (9.0)	**<0.001**	0.941
	CDSS	64.3 (8.1)	68.2 (7.9)	**<0.001**	
	*p* ^1^	1.000	0.885		
Fat (%) mean (SD)	control	29.2 (6.9)	31.9 (7.5)	**<0.001**	0.640
	CDSS	29.6 (7.0)	29.4 (7.1)	0.431	
	*p* ^1^	0.862	0.289		
Triacylglycerols (mg/dL) ^a^ median (IQR)	control	100.0 (51.0)	89.5 (69.5)	0.552	0.341
	CDSS	67.0 (52.0)	59.0 (42.5)	**0.001**	
	*p* ^1^	0.435	0.061		
Glucose (mg/dL) mean (SD)	control	97.1 (21.3)	95.4 (20.1)	0.212	0.679
	CDSS	101.3 (6.8)	95.2 (4.8)	**<0.001**	
	*p* ^1^	0.415	0.955		
Total Cholesterol (mg/dL) mean (SD)	control	192.6 (38.3)	195.0 (37.4)	0.687	0.127
	CDSS	182.7 (41.0)	167.9 (39.6)	**0.002**	
	*p* ^1^	0.435	0.032		
LDL (mg/dL) mean (SD)	control	114.7 (33.3)	115.5 (34.7)	0.870	0.702
	CDSS	115.9 (40.7)	105.8 (35.3)	**0.002**	
	*p* ^1^	0.923	0.387		
HDL (mg/dL) ^a^ median (IQR)	control	55.0 (15.0)	58.0 (26.8)	0.095	0.433
	CDSS	63.0 (15.0)	71.0 (9.8)	0.035	
	*p* ^1^	0.174	0.583		
CRP (m/L) ^a^ median (IQR)	control	0.8 (0.2)	0.7 (0.2)	0.082	0.096
	CDSS	0.7 (0.1)	0.7 (0.1)	0.705	
	*p* ^1^	0.086	0.478		

Quantitative variables are expressed as mean (SD) in the case of a normal distribution and as median (IQR) in the case of a non-normal distribution, *p*^1^: *p* value for group comparison (independent sample t-test or Mann–Whitney U test), *p*^2^: *p* value for time effect (paired-samples t-test or Wilcoxon signed-rank test), *p*^3^: between-subjects effect of treatment, repeated measures ANOVA. ^a^ Nonparametric test (Mann–Whitney U test or Wilcoxon signed-rank test) was applied, and log transformations were done before performing a repeated measures ANOVA. *p* < 0.0031 is considered statistically significant with Bonferroni correction. CDSS, clinical decision support system; LDL, low density lipoprotein; HDL, high density lipoprotein; and CRP, C-reactive protein.

**Table 4:** Dietary intake at baseline and follow-up.

Characteristics	Group	Baseline (*n* = 20)	Follow-Up (*n* = 20)	*p* ^2^	*p* ^3^
MetDietScore ^a^ median (IQR)	control	32.0 (3.8)	34.0 (2.0)	0.007	**<0.001**
	CDSS	35.0 (3.8)	38.0 (2.8)	**<0.001**	
	*p* ^1^	**<0.001**	**<0.001**		
Fibre Intake (g) mean (SD)	control	20.1 (3.8)	20.9 (3.4)	0.075	0.978
	CDSS	17.9 (3.8)	23.2 (4.4)	**<0.001**	
	*p* ^1^	0.072	0.074		
Protein Intake (%) ^a^ median (IQR)	control	18.0 (5.0)	19.5 (2.8)	0.063	0.713
	CDSS	21.0 (4.0)	20.0 (3.0)	0.503	
	*p* ^1^	0.231	0.355		
Carbohydrates (%) ^a^ median (IQR)	control	54.0 (8.0)	52.0 (5.8)	0.777	0.374
	CDSS	54.0 (6.0)	52.0 (3.5)	0.023	
	*p* ^1^	0.429	0.583		
Fat Intake (%) ^a^ median (IQR)	control	29.0 (4.0)	29.0 (3.0)	0.419	0.051
	CDSS	24.0 (6.0)	28.0 (3.8)	0.009	
	*p* ^1^	0.011	0.779		
Energy Intake (kcal) ^a^ median (IQR)	control	2300.0 (800.0)	2375.0 (900.0)	0.715	**<0.001**
	CDSS	2000.0 (100.0)	2000.0 (150.0)	0.707	
	*p* ^1^	**<0.001**	0.017		

Quantitative variables are expressed as mean (SD) in the case of a normal distribution and as median (IQR) in the case of a non-normal distribution, *p*^1^: *p* value for group comparison (independent sample t-test or Mann–Whitney U test), *p*^2^: *p* value fortime effect (paired-samples t-test or Wilcoxon signed-rank test), *p*^3^: between-subjects effect of treatment, repeated measures ANOVA. ^a^ Nonparametric test (Mann–Whitney U test or Wilcoxon signed-rank test) was applied, and log transformations were done before performing a repeated measures ANOVA. *p* < 0.0031 is considered statistically significant with Bonferroni correction. CDSS, clinical decision support system.

**Table 5:** HADS at baseline and follow-up.

Characteristics	Group	Baseline (*n* = 20)	Follow-Up (*n* = 20)	*p* ^2^	*p* ^3^
HADS (anxiety) mean (SD)	control	8.7 (4.3)	7.1 (3.1)	0.036	0.048
	CDSS	8.2 (4.2)	3.5 (2.2)	**<0.001**	
	*p* ^1^	0.711	**<0.001**		
HADS (depression) ^a^ median (IQR)	control	3.0 (2.8)	1.0 (1.0)	**0.002**	0.006
	CDSS	3.0 (5.5)	3.0 (4.5)	0.054	
	*p* ^1^	0.968	0.192		

Quantitative variables are expressed as mean (SD) in the case of a normal distribution and as median (IQR) in the case of a non-normal distribution, *p*^1^: *p* value for group comparison (independent sample t-test or Mann–Whitney U test), *p*^2^: *p* value fortime effect (paired-samples t-test or Wilcoxon sign-rank test), *p*^3^: between-subjects effect of treatment, repeated measures ANOVA. ^a^ Nonparametric tests (Mann–Whitney U test or Wilcoxon sign-rank test) was applied, and log transformations were done before performing a repeated measures ANOVA. *p* < 0.0031 is considered statistically significant with Bonferroni correction. CDSS, clinical decision support system; and HADS, Hospital Anxiety and Depression Scale.

## Data Availability

The data presented in this study are available on request from the corresponding author.

## Supplementary Materials

The following supporting information can be downloaded at: https://www.mdpi.com/article/10.3390/nu15020432/s1, Table S1: Standard diet 2200 kcal pregnancy.
