# Supplementary material for: Improving Adherence to the Mediterranean Diet in Early Pregnancy Using a Clinical Decision Support System; A Randomised Controlled Clinical Trial
Source: Nutrients. 2023 Jan 14;15(2):432. doi: 10.3390/nu15020432 (PMC9866975; doi:10.3390/nu15020432)
Supplement: Supplementary file 1 [file nutrients-15-00432-s001.zip › nutrients-2109371-Supplementary Table S1.pdf]

STANDARD DIET 2200 KCAL
PREGNANCY

"WHITE" MEAT	MEALS	MENU 1 TIME WEEKLY
	**BREAKFAST**	Oats cream (quaker): Fresh milk (1) 0-3.49% fat (1 cup) + oat flakes (3 oz.)+ 1 banana (4 oz.)
	**SNACK**	Apple (1 small, 4 oz.) + Pear (3 oz.)+ 1 cereals bar (high in fiber, low in fat, ~90kcal)
	**LUNCH**	Seasonal salad (1) or vegetable soup + chicken, without its fat crust, cooked with homemade mustard sauce e.g. (5 oz.) + boiled corn (4 oz) + wild rice, cooked (2 cups, 11oz.)
	**SNACK**	Fresh orange juice (1 cup) + almonds, or any other type of nuts, fresh (0.5 oz.)
	**DINNER**	Seasonal salad (1) + couscous, cooked (1 ½ cup) + 2 oz. Feta cheese

"RED" MEAT	MEALS	MENU 2 TIMES WEEKLY
	**BREAKFAST**	Fresh milk (1) 0- 3.49% fat (1 cup) + 2 sl. Whole wheat bread (1oz. each) + ½ tbs tachini + ½ tsp honey+ Kiwi (2 small)
	**SNACK**	Blended juice (1 medium banana, 4.2. oz., 1 small apple 4.2 oz., 1 medium orange) +1 sandwich (2 whole wheat bread slices for 2 oz., 1 oz. cheese medium in fat, low fat cold cuts (0.5 oz.)
	**LUNCH**	Seasonal salad (1) or vegetable soup + beef steak, without visible fat, cooked (or any other food), low in calories (5.2 oz.)+ cooked potato (4 small, 12 oz.)
	**SNACK**	Apple (1 small, 4.2 oz.)+ Raisins (0.5 oz.)
	**DINNER**	1 sandwich, whole wheat (3 oz. bread) + 1 oz low fat cheese λιπαρά + 0.5 oz ham + olives+ green vegetables

FISH	MEALS	MENU 1 TIME WEEKLY
	**BREAKFAST**	Fresh milk (1) 0-3.49% fat (1 cup)+ oat flakes (3 oz.)+ ½ banana (2 oz.)
	**SNACK**	Fresh orange juice (1 ½ cups)
	**LUNCH**	Seasonal salad (1)or vegetable soup + fish (5 oz.) + Baked potatoes (12 oz.)
	**SNACK**	Grapes (~20, 1.5 oz.) + whole wheat bread (1 slice, 1 oz.) with 1tsp tachini/ margarine + a little honey
	**DINNER**	Seasonal salad (1) + homemade french fries (8 oz.) + 1 fried egg + Kiwis (2 small, 2.5 oz each)

GREEK-OLIVE OIL	MEALS	MENU 1 TIME WEEKLY
	**BREAKFAST**	Fresh orange juice (1 ½ cup) + 1 toast (2 slices whole wheat bread, 2 oz.) + 1 slice low/semi- fat cheese (1 oz.) + 1 slice low fat ham (1 oz.)
	**SNACK**	Pear (1 medium, 6 oz.) + orange (1 medium, 4 oz.)
	**LUNCH**	Peas with olive and tomato sauce (or other cooked vegetables) (1 restaurant serving 12 oz.) + feta cheese (2 oz.) + bread (3 slices, 3 oz.)
	**SNACK**	Yogurt, semi- fat (2-3.49%) (1cup, 7 oz.) + muesli (1 oz.) +apple with skin (1 small, 4 oz.) + 1 tbs linseed, blended
	**DINNER**	Seasonal salad(1) + cooked fish (3 oz.) + wild rice, cooked (8 oz.)

	MEALS	MENU 1 TIME WEEKLY
L E G U M E S	BREAKFAST	Yogurt, semi- fat (2-3.49%) (1cup, 7 oz.) + oat flakes (2 oz) + kiwis (2 servings, 5 oz.) + banana (1 medium, 4 oz.) + 1tbs. linseed, blended
	SNACK	Blended juice (1 medium banana, 1 medium orange, 1 small apple)
	LUNCH	Seasonal salad (1) + lentil soup with tomato sauce (1 serving, 10oz.) + feta cheese (2 oz.) + bread (2 slices, 2 oz.)
	SNACK	Apple (1 small, 4.2 oz.) + Bread sticks, whole wheat (2 small, 1 oz.)
	DINNER	Seasonal salad (1) + chicken, without its fat crust, cooked (2 oz.) + wild rice, cooked (2 cups, 11 oz.)

	MEALS	MENU 1 TIME WEEKLY
S T A R C H	BREAKFAST	Fresh orange juice (1 ½ cup) + 1 toast (2 slices whole wheat bread, 2 oz.) + 1 slice low/semi- fat cheese (1 oz.) + omelet of 1 egg (2.5 oz.)
	SNACK	Apple (4 oz) + grapes (1.8 oz.) + fresh almonds or other nuts (0.5 oz.)
	LUNCH	Seasonal salad (1) + spaghetti, cooked (2 cups, 9 oz.) + homemade sauce + 4 tbs grinded parmesan
	SNACK	Yogurt, semi- fat (2-3.49%) (7 oz) + 1 banana, medium (4oz) + 2 whole wheat rusks (1 oz.)
	DINNER	Seasonal salad (1) or vegetable soup + legumes (1/2 cup, 3.5 oz.) + bread (1 regular slices, about 1 oz.) + 2 oz. feta cheese

**Nutritional Facts:**	
**Energy (Kcal)**	2200
**Proteins**	3.4 oz (17.4%)
**Carbohydrates**	10.4 oz (53.5%)
**Fats**	2.6 oz (30.3%)

TIPS:

(1) **Seasonal salad** (from non-starchy vegetables) may be raw or boiled. As for the total amount of olive oil (for lunch and dinner), it should be equal to 2 tablespoons (1 tablespoon= 3 teaspoons).
In meals such as meat, rice, sauces and purees try to add vegetables, so that the daily consumption of vegetables is increased (e.g. carrot puree, tomato sauce, broccoli bites)

Quantities are based on cooked food, normally prepared with ½ - 1 tbs olive oil per food serving.

Feel free to consult the unit “Food Groups” in order to make changes and to develop your own diet based on your own nutritional preferences.
Meat, fish and legumes belong in the group of proteins
Rice, potatoes and bread belong in the group of carbohydrates
Olive oil and nuts belong in the group of fat.
